# Supplementary material for: Identification of genes that differentiate Mannheimia haemolytica genotypes 1 and 2 using a pangenome approach
Source: PLoS One. 2025 Oct 16;20(10):e0325338. doi: 10.1371/journal.pone.0325338 (PMC12530517; doi:10.1371/journal.pone.0325338)
Supplement: S1 Fig — G1 and G2 clusters are indicated. (PDF) [file pone.0325338.s002.pdf]

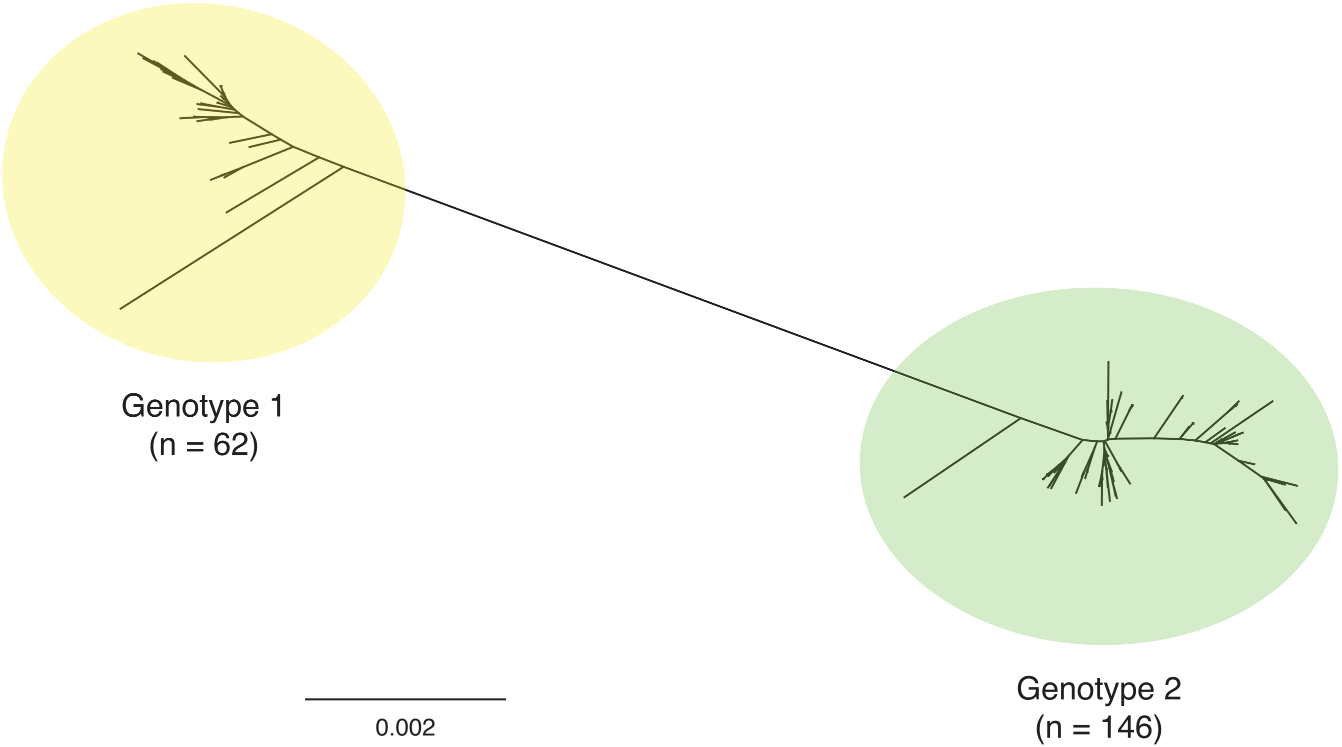

S1 Figure. Phylogenetic tree of study genomes created using MASH with the ani command in PanTools. G1 and G2 clusters are indicated.
